# Dichlorido{2-[(4-iodo­phen­yl)imino­meth­yl]pyridine-κ^2^ *N*,*N*′}copper(II)

**DOI:** 10.1107/S1600536809011684

**Published:** 2009-04-22

**Authors:** Ali Mahmoudi, Mehdi Khalaj, Shan Gao, Seik Weng Ng, Mahmoud Mohammadgholiha

**Affiliations:** a Department of Chemistry, Islamic Azad University, Karaj Branch, Karaj, Iran; b Department of Chemistry, Islamic Azad University, Buinzahra Branch, Qazvin, Iran; c School of Chemistry and Materials Science, Heilongjiang University, Harbin 150080, People’s Republic of China; d Department of Chemistry, University of Malaya, 50603 Kuala Lumpur, Malaysia

## Abstract

The Cu^II^ atom in the title complex, [CuCl_2_(C_12_H_9_IN_2_)], has a square-planar coordination being *N*,*N*′-chelated by the Schiff base ligand, and by two Cl atoms. The geometry is distorted towards square pyramidal owing to a long Cu⋯Cl inter­action of 2.941 (1) Å. This results in the formation of a zigzag chain structure propagating in the *c*-axis direction.

## Related literature

For background to the synthesis and structure of metal complexes of diimines, see: Yamada (1999 ▶). For the structure of the zinc chloride complex of the same ligand, see: Dehghanpour *et al.* (2007 ▶).
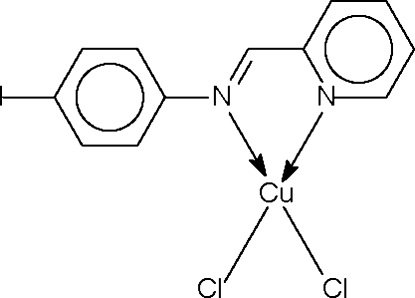


## Experimental

### 

#### Crystal data

[CuCl_2_(C_12_H_9_IN_2_)]
*M* *_r_* = 442.55
Monoclinic, 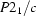

*a* = 12.2721 (5) Å
*b* = 15.2159 (5) Å
*c* = 7.4709 (2) Å
β = 94.8913 (10)°
*V* = 1389.97 (8) Å^3^
*Z* = 4
Mo *K*α radiation
μ = 4.16 mm^−1^
*T* = 295 K
0.31 × 0.25 × 0.17 mm

#### Data collection

Rigaku RAXIS-RAPID diffractometer
Absorption correction: multi-scan (*ABSCOR*; Higashi, 1995 ▶) *T*_min_ = 0.359, *T*_max_ = 0.538 (expected range = 0.329–0.493)
21713 measured reflections
3166 independent reflections
2650 reflections with *I* > 2σ(*I*)
*R*_int_ = 0.037

#### Refinement

*R*[*F* ^2^ > 2σ(*F* ^2^)] = 0.028
*wR*(*F* ^2^) = 0.079
*S* = 1.05
3166 reflections
163 parameters
H-atom parameters constrained
Δρ_max_ = 0.88 e Å^−3^
Δρ_min_ = −0.51 e Å^−3^

### 

Data collection: *RAPID-AUTO* (Rigaku, 1998 ▶); cell refinement: *RAPID-AUTO*; data reduction: *CrystalStructure* (Rigaku/MSC, 2002 ▶); program(s) used to solve structure: *SHELXS97* (Sheldrick, 2008 ▶); program(s) used to refine structure: *SHELXL97* (Sheldrick, 2008 ▶); molecular graphics: *X-SEED* (Barbour, 2001 ▶); software used to prepare material for publication: *SHELXL97*.

## Supplementary Material

Crystal structure: contains datablocks I, 9R. DOI: 10.1107/S1600536809011684/su2104sup1.cif

Structure factors: contains datablocks I. DOI: 10.1107/S1600536809011684/su2104Isup2.hkl

Additional supplementary materials:  crystallographic information; 3D view; checkCIF report

## Figures and Tables

**Fig. 1.**
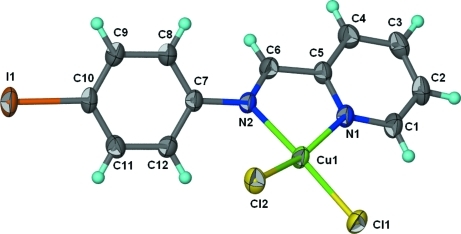
A view of the structure of the title complex, with displacement ellipsoids drawn at the 50% probability level [H atoms are represented as spheres of arbitrary radius].

**Fig. 2.**
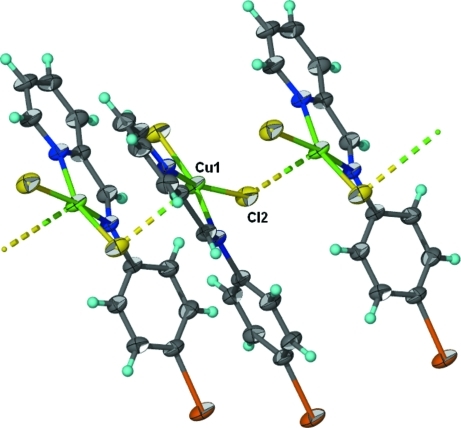
A view of the Cu···Cl2i bridged zigzag bridged chain structure of the title compound [Symmetry operations (i) = x, 1.5-y, -0.5+z]

## supplementary crystallographic information

### Comment

The structure of the title complex is illustrated in Fig. 1, and the geometrical parameters are available in the archived CIF. The complex was prepared by the reaction of copper chloride with the Schiff base ligand 2-[(4-iodophenyl)iminomethyl]pyridine (Yamada, 1999). The ligand is slightly twisted with the benzene ring and pyridine ring being inclined to one another by 26.07813)°.

A long Cu···Cl2^i^ [symmetry operation (i) = x, 1.5-y, 0.5+z] interaction of 2.941 (1) Å, leads the formation of a zigzag chain propagating in the c direction (Fig. 2). This situation is different to that observed in the ZnCl_2_ complex of the same ligand, which is mononuclear (Dehghanpour *et al.*,  2007).

### Experimental

To a solution of 2-[(4-iodophenyl)iminomethyl]pyridine (30.8 mg, 0.1 mmol) in 20 ml acetonitrile was added copper(II) chloride (13.4 mg, 0.1 mmol). The mixture was heated to dissolve the reactants and then the solution was filtered and the volume reduced under vacuum to ca. 5 ml. Diffusion of diethyl ether vapor into the solution gave green crystals of the title complex. The crystals were collected and washed with diethylether-dichloromethane (9:1 *v*/*v*). Yield 75%. CHN elemental analysis: Calc. for C_12_H_9_Cl_2_CuIN_2_: C 32.57, H 2.05, N 6.33%; found: C 32.54, H 2.07, N 6.35%.

### Refinement

The H-atoms were placed in calculated positions [C—H 0.93 Å] and treated as riding atoms [*U*_iso_(H) = 1.2*U*_eq_(C)].

### Crystal data

**Table:**

[CuCl_2_(C_12_H_9_IN_2_)]	*F*(000) = 844
*M**_r_* = 442.55	*D*_x_ = 2.115 Mg m^−^^3^
Monoclinic, *P*2_1_/*c*	Mo *K*α radiation, λ = 0.71073 Å
Hall symbol: -P 2ybc	Cell parameters from 13838 reflections
*a* = 12.2721 (5) Å	θ = 3.1–27.5°
*b* = 15.2159 (5) Å	µ = 4.16 mm^−^^1^
*c* = 7.4709 (2) Å	*T* = 295 K
β = 94.8913 (10)°	Block, green
*V* = 1389.97 (8) Å^3^	0.31 × 0.25 × 0.17 mm
*Z* = 4	

### Data collection

**Table:**

Rigaku RAXIS-RAPID diffractometer	3166 independent reflections
Radiation source: fine-focus sealed tube	2650 reflections with *I* > 2σ(*I*)
graphite	*R*_int_ = 0.037
Detector resolution: 10.000 pixels mm^-1^	θ_max_ = 27.5°, θ_min_ = 3.1°
ω scans	*h* = −15→15
Absorption correction: multi-scan (*ABSCOR*; Higashi, 1995)	*k* = −19→19
*T*_min_ = 0.359, *T*_max_ = 0.538	*l* = −9→9
21713 measured reflections	

### Refinement

**Table:**

Refinement on *F*^2^	Primary atom site location: structure-invariant direct methods
Least-squares matrix: full	Secondary atom site location: difference Fourier map
*R*[*F*^2^ > 2σ(*F*^2^)] = 0.028	Hydrogen site location: inferred from neighbouring sites
*wR*(*F*^2^) = 0.079	H-atom parameters constrained
*S* = 1.05	*w* = 1/[σ^2^(*F*_o_^2^) + (0.0447*P*)^2^ + 0.6865*P*] where *P* = (*F*_o_^2^ + 2*F*_c_^2^)/3
3166 reflections	(Δ/σ)_max_ = 0.001
163 parameters	Δρ_max_ = 0.88 e Å^−^^3^
0 restraints	Δρ_min_ = −0.51 e Å^−^^3^

### Fractional atomic coordinates and isotropic or equivalent isotropic displacement parameters (Å^2^)

**Table:**

	*x*	*y*	*z*	*U*_iso_*/*U*_eq_	
I1	−0.001646 (16)	0.649922 (16)	0.55181 (3)	0.05798 (10)	
Cu1	0.60873 (3)	0.72391 (2)	0.87653 (5)	0.03844 (11)	
Cl1	0.72032 (6)	0.82416 (5)	1.01818 (12)	0.05137 (19)	
Cl2	0.51519 (6)	0.82558 (5)	0.71178 (11)	0.04616 (18)	
N1	0.70612 (18)	0.62040 (15)	0.9438 (3)	0.0375 (5)	
N2	0.50252 (17)	0.62295 (15)	0.7935 (3)	0.0334 (4)	
C1	0.8056 (2)	0.6212 (2)	1.0289 (4)	0.0469 (7)	
H1	0.8344	0.6738	1.0752	0.056*	
C2	0.8676 (2)	0.5448 (2)	1.0503 (5)	0.0520 (8)	
H2	0.9368	0.5463	1.1114	0.062*	
C3	0.8259 (3)	0.4672 (2)	0.9809 (5)	0.0489 (7)	
H3	0.8677	0.4162	0.9895	0.059*	
C4	0.7210 (3)	0.4657 (2)	0.8980 (4)	0.0443 (6)	
H4	0.6902	0.4135	0.8530	0.053*	
C5	0.6629 (2)	0.54354 (18)	0.8832 (4)	0.0365 (6)	
C6	0.5499 (2)	0.54841 (18)	0.8064 (4)	0.0378 (6)	
H6	0.5128	0.4977	0.7672	0.045*	
C7	0.3893 (2)	0.62851 (18)	0.7297 (4)	0.0350 (5)	
C8	0.3398 (2)	0.5679 (2)	0.6095 (4)	0.0446 (6)	
H8	0.3814	0.5233	0.5643	0.054*	
C9	0.2287 (2)	0.5736 (2)	0.5565 (4)	0.0479 (7)	
H9	0.1957	0.5323	0.4775	0.057*	
C10	0.1677 (2)	0.64099 (19)	0.6219 (4)	0.0417 (6)	
C11	0.2166 (2)	0.70219 (19)	0.7420 (4)	0.0422 (6)	
H11	0.1753	0.7475	0.7852	0.051*	
C12	0.3266 (2)	0.69542 (18)	0.7964 (4)	0.0385 (6)	
H12	0.3591	0.7357	0.8781	0.046*	

### Atomic displacement parameters (Å^2^)

**Table:**

	*U*^11^	*U*^22^	*U*^33^	*U*^12^	*U*^13^	*U*^23^
I1	0.02714 (12)	0.06908 (18)	0.07514 (18)	0.00149 (8)	−0.01061 (10)	0.00131 (11)
Cu1	0.02850 (18)	0.03088 (18)	0.0536 (2)	0.00103 (12)	−0.00989 (14)	−0.00045 (14)
Cl1	0.0377 (4)	0.0440 (4)	0.0702 (5)	−0.0064 (3)	−0.0082 (3)	−0.0106 (4)
Cl2	0.0382 (4)	0.0366 (3)	0.0624 (4)	0.0042 (3)	−0.0032 (3)	0.0115 (3)
N1	0.0270 (11)	0.0373 (11)	0.0472 (12)	0.0010 (9)	−0.0036 (9)	0.0017 (11)
N2	0.0265 (11)	0.0321 (10)	0.0404 (11)	0.0000 (8)	−0.0033 (8)	0.0008 (9)
C1	0.0306 (14)	0.0500 (16)	0.0578 (17)	0.0032 (12)	−0.0087 (12)	−0.0016 (15)
C2	0.0279 (14)	0.0624 (19)	0.0640 (19)	0.0063 (13)	−0.0055 (13)	0.0086 (16)
C3	0.0358 (16)	0.0470 (16)	0.0640 (18)	0.0124 (12)	0.0054 (13)	0.0129 (15)
C4	0.0390 (15)	0.0367 (14)	0.0570 (17)	0.0040 (11)	0.0022 (13)	0.0056 (13)
C5	0.0287 (13)	0.0353 (13)	0.0449 (14)	0.0019 (10)	−0.0002 (10)	0.0041 (12)
C6	0.0315 (13)	0.0325 (13)	0.0484 (15)	−0.0023 (10)	−0.0027 (11)	0.0010 (12)
C7	0.0264 (12)	0.0349 (12)	0.0429 (13)	0.0000 (10)	−0.0022 (10)	0.0020 (12)
C8	0.0304 (14)	0.0513 (17)	0.0513 (16)	0.0015 (12)	−0.0014 (11)	−0.0108 (14)
C9	0.0331 (15)	0.0581 (18)	0.0507 (16)	−0.0033 (13)	−0.0069 (12)	−0.0096 (15)
C10	0.0244 (13)	0.0492 (16)	0.0503 (16)	−0.0011 (11)	−0.0046 (11)	0.0049 (13)
C11	0.0320 (14)	0.0387 (14)	0.0556 (16)	0.0054 (11)	0.0017 (11)	0.0012 (13)
C12	0.0326 (14)	0.0360 (13)	0.0457 (14)	−0.0010 (11)	−0.0036 (11)	−0.0015 (12)

### Geometric parameters (Å, °)

**Table:**

I1—C10	2.104 (3)	C4—C5	1.383 (4)
Cu1—N1	2.015 (2)	C4—H4	0.9300
Cu1—N2	2.075 (2)	C5—C6	1.457 (4)
Cu1—Cl1	2.253 (1)	C6—H6	0.9300
Cu1—Cl2	2.233 (1)	C7—C8	1.390 (4)
N1—C1	1.328 (4)	C7—C12	1.393 (4)
N1—C5	1.346 (4)	C8—C9	1.389 (4)
N2—C6	1.274 (4)	C8—H8	0.9300
N2—C7	1.433 (3)	C9—C10	1.384 (4)
C1—C2	1.391 (4)	C9—H9	0.9300
C1—H1	0.9300	C10—C11	1.393 (4)
C2—C3	1.371 (5)	C11—C12	1.380 (4)
C2—H2	0.9300	C11—H11	0.9300
C3—C4	1.380 (4)	C12—H12	0.9300
C3—H3	0.9300		
			
N1—Cu1—N2	80.79 (9)	N1—C5—C4	122.1 (3)
N1—Cu1—Cl2	160.96 (7)	N1—C5—C6	115.0 (2)
N1—Cu1—Cl1	95.07 (7)	C4—C5—C6	122.8 (3)
N2—Cu1—Cl1	169.40 (7)	N2—C6—C5	119.2 (2)
N2—Cu1—Cl2	93.89 (6)	N2—C6—H6	120.4
Cl1—Cu2—Cl1	93.05 (3)	C5—C6—H6	120.4
C1—N1—C5	119.3 (2)	C8—C7—C12	119.5 (2)
C1—N1—Cu1	127.9 (2)	C8—C7—N2	122.2 (2)
C5—N1—Cu1	112.76 (17)	C12—C7—N2	118.3 (2)
C6—N2—C7	120.1 (2)	C7—C8—C9	120.5 (3)
C6—N2—Cu1	111.44 (18)	C7—C8—H8	119.8
C7—N2—Cu1	128.50 (17)	C9—C8—H8	119.8
N1—C1—C2	121.2 (3)	C10—C9—C8	119.5 (3)
N1—C1—H1	119.4	C10—C9—H9	120.3
C2—C1—H1	119.4	C8—C9—H9	120.3
C3—C2—C1	119.6 (3)	C9—C10—C11	120.4 (3)
C3—C2—H2	120.2	C9—C10—I1	120.9 (2)
C1—C2—H2	120.2	C11—C10—I1	118.7 (2)
C2—C3—C4	119.2 (3)	C12—C11—C10	119.8 (3)
C2—C3—H3	120.4	C12—C11—H11	120.1
C4—C3—H3	120.4	C10—C11—H11	120.1
C3—C4—C5	118.5 (3)	C11—C12—C7	120.3 (3)
C3—C4—H4	120.8	C11—C12—H12	119.8
C5—C4—H4	120.8	C7—C12—H12	119.8
			
N2—Cu1—N1—C1	−175.5 (3)	C3—C4—C5—N1	1.4 (5)
Cl2—Cu1—N1—C1	109.5 (3)	C3—C4—C5—C6	−176.8 (3)
Cl1—Cu1—N1—C1	−5.4 (3)	C7—N2—C6—C5	−175.7 (2)
N2—Cu1—N1—C5	8.14 (19)	Cu1—N2—C6—C5	3.6 (3)
Cl2—Cu1—N1—C5	−66.8 (3)	N1—C5—C6—N2	3.3 (4)
Cl1—Cu1—N1—C5	178.30 (18)	C4—C5—C6—N2	−178.3 (3)
N1—Cu1—N2—C6	−6.3 (2)	C6—N2—C7—C8	−29.4 (4)
Cl2—Cu1—N2—C6	155.25 (19)	Cu1—N2—C7—C8	151.3 (2)
Cl1—Cu1—N2—C6	−74.0 (4)	C6—N2—C7—C12	148.2 (3)
N1—Cu1—N2—C7	172.9 (2)	Cu1—N2—C7—C12	−31.0 (3)
Cl2—Cu1—N2—C7	−25.5 (2)	C12—C7—C8—C9	−0.2 (4)
Cl1—Cu1—N2—C7	105.3 (4)	N2—C7—C8—C9	177.4 (3)
C5—N1—C1—C2	2.6 (5)	C7—C8—C9—C10	1.1 (5)
Cu1—N1—C1—C2	−173.5 (2)	C8—C9—C10—C11	−0.8 (5)
N1—C1—C2—C3	0.6 (5)	C8—C9—C10—I1	−178.1 (2)
C1—C2—C3—C4	−2.9 (5)	C9—C10—C11—C12	−0.2 (5)
C2—C3—C4—C5	1.9 (5)	I1—C10—C11—C12	177.1 (2)
C1—N1—C5—C4	−3.7 (4)	C10—C11—C12—C7	1.0 (4)
Cu1—N1—C5—C4	173.0 (2)	C8—C7—C12—C11	−0.8 (4)
C1—N1—C5—C6	174.7 (3)	N2—C7—C12—C11	−178.6 (3)
Cu1—N1—C5—C6	−8.6 (3)		
